# Combination of NOS- and PDK-Inhibitory Activity: Possible Way to Enhance Antitumor Effects

**DOI:** 10.3390/ijms23020730

**Published:** 2022-01-10

**Authors:** Marina Filimonova, Anna Shitova, Olga Soldatova, Ljudmila Shevchenko, Alina Saburova, Tatjana Podosinnikova, Valentina Surinova, Petr Shegay, Andrey Kaprin, Sergey Ivanov, Alexander Filimonov

**Affiliations:** 1 Personalized Medicine Centre, Almazov National Medical Research Centre, 197341 Saint Petersburg, Russia; annaredrose@mail.ru (A.S.); 89208861291@mail.ru (O.S.); schew.ludmila@yandex.ru (L.S.); val_suriniva@mail.ru (V.S.); filimonov_alex@mail.ru (A.F.); 2 A. Tsyb Medical Radiological Research Center—Branch of the National Medical Research Radiological Center of the Ministry of Health of the Russian Federation, 249036 Obninsk, Russia; alinasamsonova.515@gmail.com (A.S.); tanchitto@mail.ru (T.P.); oncourolog@gmail.com (S.I.); 3 National Medical Research Radiological Center of the Ministry of Health of the Russian Federation, 249036 Obninsk, Russia; dr.shegai@mail.ru (P.S.); kaprin@mail.ru (A.K.)

**Keywords:** NOS-inhibitor, PDK-inhibitor, antitumor effect, hypoxic resistance, Erlich carcinoma

## Abstract

We have previously demonstrated a high antitumor potential of NOS inhibitor T1023 (1-isobutanoyl-2-isopropylisothiourea hydrobromide): antitumor antiangiogenic activity in several animal tumor models and its ability to synergistically enhance the antitumor effects of bevacizumab, cyclophosphamide and γ-radiation. At the same time, rather rapid adaptation of experimental neoplasias to T1023 treatment was often observed. We attempted to enhance the antitumor activity of this NOS inhibitor by supplementing its molecular structure with a PDK-inhibiting fragment, dichloroacetate (DCA), which is capable of hypoxia-oriented toxic effects. We synthesized compound T1084 (1-isobutanoyl-2-isopropylisothiourea dichloroacetate). Its toxic properties, NOS-inhibiting and PDK-inhibiting activity in vivo, and antitumor activity on the mouse Ehrlich carcinoma model (SEC) were investigated in compare with T1023 and Na-DCA. We found that the change of the salt-forming acid from HBr to DCA does not increase the toxicity of 1-isobutanoyl-2-isopropylisothiourea salts, but significantly expands the biochemical and anti-tumor activity. New compound T1084 realizes in vivo NOS-inhibiting and PDK-inhibiting activity, quantitatively, at the level of the previous compounds, T1023 and Na-DCA. In two independent experiments on SEC model, a pronounced synergistic antitumor effect of T1084 was observed in compare with T1023 and Na-DCA at equimolar doses. There were no signs of SEC adaptation to T1084 treatment, while experimental neoplasia rapidly desensitized to the separate treatment of both T1023 and Na-DCA. The totality of the data obtained indicates that the combination of antiangiogenic and hypoxia-oriented toxic effects (in this case, within the molecular structure of the active substance) can increase the antitumor effect and suppress the development of hypoxic resistance of neoplasias. In general, the proposed approach can be used for the design of new anticancer agents.

## 1. Introduction

A significant amount of data indicating the active and multilateral participation of endogenous nitric oxide (NO) in angiogenesis and tumor progression has been gained in recent decades [1,2,3,4,5]. In this regard, the ability of nitric oxide synthase (NOS) inhibitors to exert an antiangiogenic effect on neoplasias [6,7,8,9,10] seems to be quite natural.

**Figure 1:**
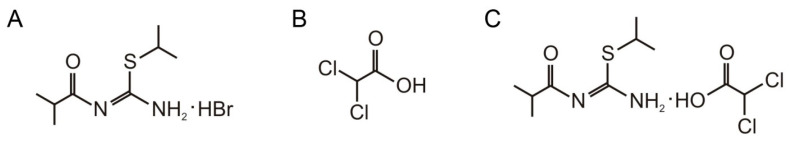
Structural formulas of the compounds studied. (**A**) 1-isobutanoyl-2-isopropylisothiourea hydrobromide (compound T1023); (**B**) dichloroacetate (DCA); (**C**) 1-isobutanoyl-2-isopropylisothiourea dichloroacetate (compound T1084).

One of them, 1-isobutanoyl-2-isopropylisothiourea hydrobromide (compound T1023; Figure 1A), is an effective competitive inhibitor of iNOS and eNOS (IC_50_ for nNOS, iNOS, eNOS: 52.3, 3.2 and 5.1 μM, respectively). Its chronic parenteral administration at doses of 30–60 mg/kg (1/9-1/5 LD_16_) causes inhibition of tumor growth and suppression of metastasis in a number of transplanted solid tumors in mice. T1023 synergistically increases the antitumor effect of bevacizumab, cyclophosphamide, or γ-irradiation [11,12,13,14]. These effects of T1023 are also realized, predominantly, by the antiangiogenic way. In immunohistochemical studies, by the 3rd–4th day from the start of T1023 administration, a pronounced (1.4–1.6 times) decrease in the content of vessels and capillaries in peritumoral zones and “hot spots” of angiogenesis of tumor nodes was observed. An increase (1.4–2 times) in the fraction of hypoxic cells (pO_2_ < 10 mm Hg), a decrease in proliferation (1.3–1.6 times), and a significant (2–3-fold) increase in apoptosis of tumor cells were registered in the tumor parenchyma [12,13].

At the same time, the antitumor efficacy of T1023 in chronic use, as a rule, decreased in the long-term development of experimental tumors (Lewis lung carcinoma, Ehrlich carcinoma, B-16 melanoma). Increased growth of neoplasia, increased proliferation, and weakened apoptosis of tumor cells developed against the background of persistent T1023-induced disorders of tumor vascularization and deep intratumoral hypoxia. This can be interpreted as a manifestation of the development of hypoxic adaptation of neoplasias, often accompanying antiangiogenic effects [15,16]. To overcome this phenomenon, it seemed advisable to use T1023 in combination with hypoxic cytotoxins, which mainly affect hypoxic cells.

One of the hypoxia-targeted cytotoxins is pyruvate dehydrogenase kinase (PDK) inhibitor—dichloroacetate (DCA; Figure 1B). DCA is able to induce apoptosis of hypoxic cells by stimulating the activity of the Krebs cycle and the mitochondrial respiratory chain. The sensitivity of a significant number of tumors to DCA administration and its ability to overcome the hypoxia-induced resistance of some neoplasias have been demonstrated [17,18,19,20].

We have assessed the combined use of T1023 and DCA (in the form of sodium salt—Na-DCA) in the model of solid Ehrlich carcinoma (SEC) in mice [21,22]. It was found that the combined use of T1023 (daily, i.p., 60 mg/kg) and Na-DCA (every other day, i.g., 162 mg/kg) causes a synergistic antitumor effect at different stages of SEC development, in comparison with their separate application. No manifestations of neoplasia adaptation to such effects were observed.

At the same time, the chemical properties of T1023 and DCA made it possible to develop a different approach to the combined use of these compounds. The NOS-inhibiting fragment of T1023, 1-isobutanoyl-2-isopropylisothiourea, is a weak base, and DCA is a fairly strong organic acid (pKa = 1.25). This made it possible to obtain a compound combining NOS-inhibiting and PDK-inhibiting fragments in the molecular structure—1-isobutanoyl-2-isopropylisothiourea dichloroacetate salt (compound T1084; Figure 1C). We managed to develop a method for obtaining T1084 and synthetically realize this compound [23].

In this work, we tried to assess the prospects of this approach to the development of polyfunctional anticancer agents. We studied the toxic and biochemical properties of compound T1084, as well as its antitumor activity in vivo in compare with precursor compounds T1023 and DCA.

## 2. Results

### 2.1. T1084: Toxicological Characteristics

The change in the salt-forming acid from HBr to DCA had no significant effect on the acute toxicity of 1-isobutanoyl-2-isopropylisothiourea salts. T1084 injection, single i.p. at doses of 150–600 mg/kg, caused intoxication in mice. Its manifestations and dynamics were similar to the effect of T1023 in toxic doses. Then, 2–3 min after T1084 administration, the animals became lethargic, inactive. At doses above 250 mg/kg, tachypnea was noted. Then, 10–20 min later, the reaction of mice to stimuli was inhibited, and then respiratory arrhythmia, impaired coordination, tremor, and clonic seizures developed. Respiratory arrest and death of animals were recorded in the first 20–60 min after T1084 administration in toxic doses. In surviving mice, the manifestations of intoxication began to subside after 1.5–2 h and completely regressed by 3–4 h after injection. A day later, and within 15 days of further observation, the surviving mice did not differ in general condition, motor activity, and body weight dynamics from healthy intact animals. Macroscopic changes in internal organs in dead and surviving mice were not observed at all doses of T1084 used.

Calculations of acute toxicity indicators showed that T1084 is somewhat less toxic than T1023; however it also corresponds to the third toxicity class, “moderately hazardous substances” (Table 1). A comparison of T1084 toxicity indicators with the corresponding indicators for T1023 shows that their difference in molar terms does not exceed 5–7%. This indicates that the differences in values expressed in mg/kg are mainly determined by the differences in molecular weights of T1023 and T1084.

### 2.2. T1084: Biochemical Properties

#### 2.2.1. NOS-Inhibitory Activity In Vivo

**Figure 2:**
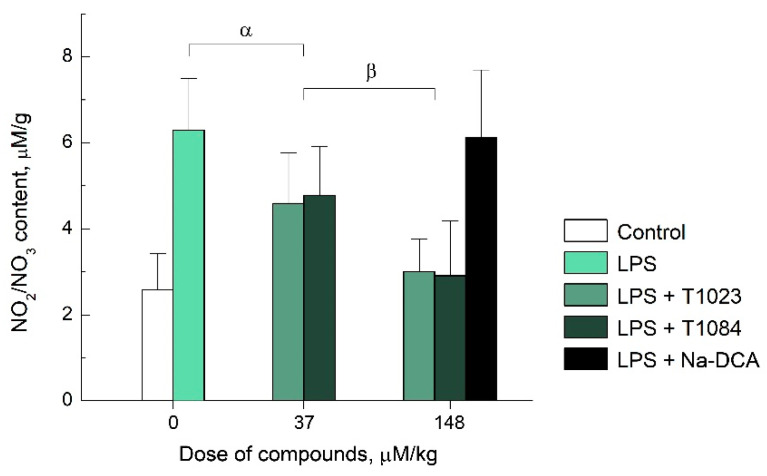
Effect of T1023 and T1084 with a single i.p. administration at equimolar doses of 37 µM/kg (10.0 and 11.8 mg/kg, respectively); and T1023, Na-DCA, T1084 at equimolar doses of 148 µM/kg (40.0, 22.2 and 47.1 mg/kg, respectively) on LPS-induced production of NO_2_/NO_3_ in liver tissue of male mice F_1_ (CBA × C57Bl/6j) (see Materials and Methods). Graphical deviations correspond to SD (*n* = 24–27 per point). Significant intergroup differences are marked with α and β. α—between the group receiving only LPS and the groups receiving T1023 and T1084 at a dose of 37 µM/kg (T1023: *p* = 0.00673; T1084: *p* = 0.00914, respectively); β—between the groups receiving NOS inhibitors at a dose of 37 and 148 µM/kg (T1023: *p* = 0.00257; T1084: *p* = 0.01846, respectively).

The study of NOS inhibitory activity in vivo showed that DCA, even at a high dose, did not have any effect on the production of NO_2_/NO_3_. At the same time, compounds T1023 and T1084 containing the NOS-inhibiting fragment were equally effective in reducing the level of NO metabolites. Both compounds, with a single i.p. administration in equimolar doses, caused a decrease in the content of stable nitric oxide metabolites (NO_2_/NO_3_) induced by LPS in mouse liver tissues (Figure 2). The severity of T1023 and T1084 activity had a distinct dose-dependent character. At a dose of 37 μM/kg, both compounds caused a moderate (by 30%) but significant (*p* = 0.006–0.009) reduction in NO_2_/NO_3_ content in LPS-induced mice. An increase in the dose of T1024 and T1084 to 148 μM/kg was accompanied also by a significant (*p* = 0.003–0.02) increase in the inhibitory effect (up to 60%). At this dose, both compounds completely suppressed LPS-induced NO hyperproduction (equality with biological control—*p* = 0.83–0.94). Moreover, at both doses used, the effects of T1023 and T1084 statistically coincided with a high probability (*p* = 0.91–0.98).

The high qualitative and quantitative similarity of T1023 and T1084 effects testifies that a change in the salt-forming acid from hydrobromic acid to DCA does not have a significant effect on the NOS-inhibiting activity of 1-isobutanoyl-2-isopropylisothiourea salts, and therefore, does not significantly modify their antiangiogenic ability.

#### 2.2.2. PDK-Inhibitory Activity In Vivo

**Figure 3:**
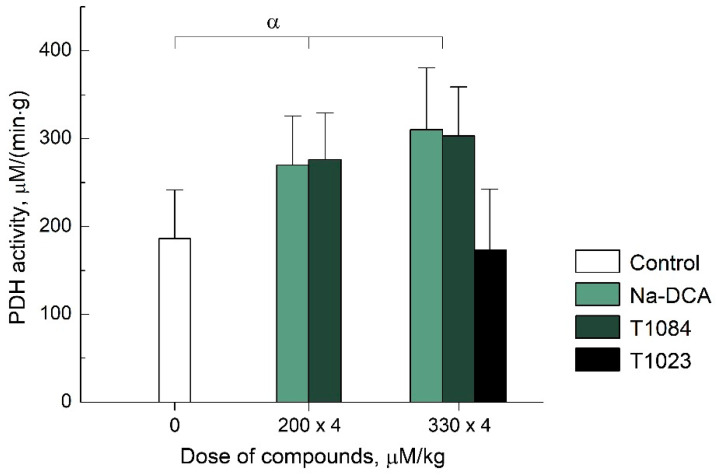
Effect of Na-DCA and T1084, 4-fold i.g. administration at equimolar doses of 200 µM/kg (30 and 63 mg/kg, respectively) and Na-DCA, T1084 and T1023, 330 µM/kg (50, 105 and 89 mg/kg, respectively), on PDH activity in male CBA mouse liver tissue (see Materials and Methods). PDH activity—rate of pyruvate oxidation in homogenate, μM/(min·g). Graphical deviations correspond to SD (*n* = 11–13 per point). α—significant differences with control animals (Na-DCA: *p* = 0.03324, *p* = 0.00409; T1084: *p* = 0.01298, *p* = 0.00442, respectively).

PDK is known to inhibit PDH activity (by phosphorylation). Therefore, the PDK activity can be estimated indirectly by the PDH activity—the rate of pyruvate oxidation. Using this approach, we conducted a comparative in vivo study of DCA (as sodium salt), T1084, and T1023 PDK inhibitory activity. T1023, even at a high dose, had no effect on PDH activity (and, accordingly, PDK activity). At the same time, the effects of Na-DCA and T1084 were significant and quantitatively similar. Both compounds, when administered four times i.g. at equimolar doses of 200 μM/kg (see Materials and Methods), caused a significant (by 50%, *p* = 0.01–0.03) increase in the rate of pyruvate oxidation in mouse liver tissues (Figure 3). At equimolar doses of 330 µM/kg Na-DCA and T1084, the PDH activity increased up to 70% (*p* = 0.004). Moreover, at both doses used, the effect of Na-DCA and T1084 on PDH activity was statistically equal (*p* = 0.4–0.7). This indicates that compound T1084 realized in vivo PDK-inhibiting activity, which does not differ significantly from that of DCA.

### 2.3. T1084: Antitumor Activity In Vivo

The results of two independent experiments in mice SEC model demonstrate a significantly modified antitumor activity in case of NOS-inhibiting fragment (1-isobutanoyl-2-isopropylisothiourea) and the PDK-inhibiting fragment (DCA) combination in the same molecular structure (T1084) in comparison with the separate effect of T1023 or sodium salt DCA (Na-DCA).

**Figure 4:**
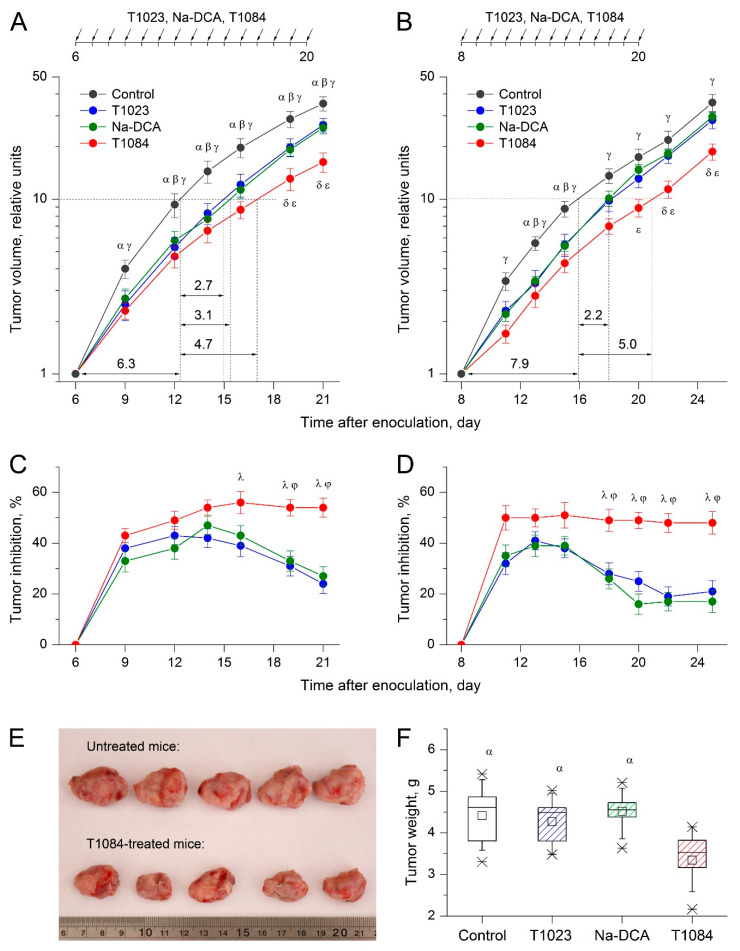
Effect of T1023, Na-DCA, and T1084 with chronic i.p. administration at equimolar doses 220 µM/kg (60.0, 33.6, and 70.7 mg/kg, respectively) on solid Ehrlich carcinoma (SEC) in female mice F_1_(CBA × C_57_Bl/6j). Data from two independent experiments ((**A**,**C**)—first; (**B**,**D**–**F**)—second). (**A**,**B**)—tumor growth curves in animal groups (*n* = 17–21 per point). Tumor volume (TV) indices for each animal are normalized to the initial volume on the day of the beginning of treatment. Graphical deviations correspond to SD. Dotted lines—estimates of the duration of tumor growth retardation (in days) by the time of a 10-fold increment in TV. Symbols—significant TV differences: α—in control mice vs. T1023-treated mice; β—in control mice vs. Na-DCA-treated mice; γ—in control mice vs. T1084-treated mice; δ—in T1084-treated mice vs. T1023-treated mice; ε—in T1084-treated mice vs. Na-DCA-treated mice. Significance of differences: α—(**A**) *p* = 0.0082, *p* = 0.0058, *p* = 0.0079, *p* = 0.0119, *p* = 0.0091, *p* = 0.0238; (**B**) *p* = 0.0054, *p* = 0.0098; β—(**A**) *p* = 0.0103, *p* = 0.0028, *p* = 0.0053, *p* = 0.0066, *p* = 0.0158; (**B**) *p* = 0.0084, *p* = 0.0068; γ—(**A**) *p* = 0.0052, *p* = 0.0006, *p* = 0.0001, *p* = 0.0001, *p* = 0.0002, *p* < 0.0001; (**B**) *p* = 0.0038, *p* = 0.0012, *p* = 0.0002, *p* = 0.0026, *p* = 0.0034, *p* = 0.0003, *p* = 0.0002; δ—(**A**) *p* = 0.0205, *p* = 0.0008; (**B**) *p* = 0.0102, *p* = 0.0094; ε—(**A**) *p* = 0.0136, *p* = 0.0112; (**B**) *p* = 0.0074, *p* = 0.0057, *p* = 0.0069. (**C**,**D**)—dynamics of tumor growth inhibition (TI) in animal groups. Graphic deviations correspond to SD. Symbols—significant TI differences: λ—in T1084-treated mice vs. T1023-treated mice; φ—in T1084-treated mice vs. Na-DCA-treated mice. Significance of differences: λ—(**C**) *p* = 0.0133, *p* = 0.0071, *p* = 0.0038, (**D**) *p* = 0.0083, *p* = 0.0070, *p* = 0.0024, *p* = 0.0045; φ—(**C**) *p* = 0.0098, *p* = 0.0051, (**D**) *p* = 0.0075, *p* = 0.0013, *p* = 0.0022, *p* = 0.0034. (**E**) Appearance of SEC tumor nodes on the 25th day after inoculation in untreated and T1084-treated mice. (**F**) Weight of SEC tumor nodes on the 25th day after inoculation in untreated, T1023-treated, DCA-treated, and T1084-treated mice (*n* = 17–20). Symbol α—significant tumor weight differences vs. T1084-treated mice. α—*p* = 0.007045, *p* = 0.0122276, *p* = 0.001116.

Thus, in both experiments, daily i.p. administration of T1023 and Na-DCA at equimolar doses of 220 μM/kg (60.0 and 33.6 mg/kg, respectively) had a similar effect on the severity and dynamics of SEC growth (Figure 4). Significant inhibition of SEC growth developed by the third to fifth day from the beginning of T1023 or Na-DCA administration (TI = 37–48%). However, in both experiments, from the 8th–10th day of T1023 and Na-DCA injections, the sensitivity of SEC to such treatment began to progressively weaken. Therefore, the inhibition of neoplasia growth decreased two-fold by the end of the observation (TI = 17–27%). As a result of such dynamics, the integral antitumor effect of T1023 and Na-DCA with their chronic use was rather limited—the time for a 10-fold increment in neoplasia volume increased 1.3–1.5 times.

Compound T1084 at the same dose of 220 μM/kg (70.7 mg/kg), daily i.p., had a stronger effect on SEC in these experiments. Its antitumor effect also developed by the third to fifth day from the start of treatment but was more pronounced (TI = 50–56%). No decrease in the sensitivity of SEC to T1084 chronic administration was observed in either experiment. The inhibition of neoplasia growth remained at a stable level (TI = 49–54%).

The significance of the difference between T1084 treatment in comparison with T1023 and Na-DCA was confirmed at the later stages of both experiments by highly significant statistical differences in tumor volume (Figure 4A,B) and in growth inhibition indices in the treated animals (Figure 4C,D). In addition, T1084 antitumor activity was quite long-term: the effect did not significantly weaken, at least for 5 days after the end of treatment (Figure 4B,D). The generalized antitumor effect of T1084 was also more pronounced: the time of a 10-fold increment in neoplasia volume increased 1.7–1.8 times, and at the terminal stage, the tumor nodes of the SEC in T1084-treated mice were objectively less (Figure 4E) and had a significantly (1.4 times) lower weight (Figure 4F) than tumors of untreated, T1023-treated, and DCA-treated animals.

## 3. Discussion

The first anti-angiogenic drug bevacizumab was approved for clinical use more than 15 years ago. To date, the arsenal of such drugs has expanded substantially, and their use in the treatment of oncological diseases has become significant. At the same time, the vast experience accumulated in this area indicates that the initial optimistic expectations have not yet been met [15,16]. Available antiangiogenic agents are effective against not all tumors. Moreover, long-term remission with antiangiogenic monotherapy is extremely rare, due to the development of neoplasia resistance to such treatment. Therefore, the clinical results of such therapy remain modest—there is a moderate improvement in overall survival. The life expectancy of patients increases by no more than 1 year.

The limited potential of antiangiogenic agents in tumor treatment is probably due to the very concept of such therapy. Induction of disorders in tumor vascularization and an increasing lack of oxygen and nutrient supply to the neoplasm determine the development of therapeutic effect—suppression of the tumor growth and metastasis. However, on the other hand, hypoxia is a powerful selective factor that modifies the biology of tumor cells. The increasing influence of this factor during antiangiogenic therapy can lead to pleotropic phenomena [24,25,26,27], making a significant contribution to the development of resistance to therapeutic effects, activation of alternative angiogenic pathways, enhancement of the pro-migration phenotype, and invasive tumor growth.

Regardless of the mechanisms of these changes (acquisition of new HIF-1α-dependent functional properties by tumor cells or enhancement of resistant clones), the cytological substrate that implements adaptation to antiangiogenic therapy is, first of all, the fraction of hypoxic tumor cells. For this reason, an expedient way to increase the capabilities of antiangiogenic agents in the treatment of tumors could be their combined use with hypoxia-oriented agents, the targets of which are, mainly, hypoxic tumor cells. We attempted to realize such a combined effect using the salt of 1-isobutanoyl-2-isopropylisothiourea dichloroacetate (compound T1084; Figure 1C), which we obtained from compound T1023 by changing the salt-forming acid HBr to DCA. Compound T1084 combines in the molecular structure the NOS-inhibiting fragment, 1-isobutanoyl-2-isopropylisothiourea, and the PDK-inhibiting fragment, DCA.

This modification of the molecular structure did not significantly affect the toxic properties of T1084. Comparative analysis shows that the indicators of acute toxicity of salts T1023 and T1084, apparently, are determined by the toxic properties of the base—1-isobutanoyl-2-isopropylisothiourea. Changing the salt-forming acid from HBr to DCA has no significant effect on the acute toxicity of T1084.

At the same time, such a change really expanded the spectrum of T1084 biochemical activity. These results indicate its both NOS-inhibiting and PDK-inhibiting abilities in vivo no less than the precursor compounds—T1023 and DCA. This polyfunctionality allows T1084 to simultaneously realize both types of pharmacological activity, antiangiogenic and hypoxia-oriented cytotoxic, that can potentially enhance its antitumor effects.

The first experimental results confirm the high antitumor potential of T1084. In repeated experiments on SEC model in mice, we observe not only a more pronounced (almost synergistic) antitumor effect of T1084 in comparison with the effects of T1023 and Na-DCA in equimolar doses. Even more significant in this study is the complete absence of signs of SEC adaptation to T1084 treatment while this neoplasia rapidly and markedly reduces the sensitivity to the separate effects of both T1023 or Na-DCA. In addition, in these experiments, T1084 proved to be a fairly safe means. Its chronic daily use did not cause intoxication and death of animals, while in the control untreated groups, 10–15% of animals died at the terminal stages.

Thus, the totality of the data obtained indicates the promising nature of our approach to the design of new polyfunctional antitumor agents capable of overcoming the hypoxic resistance of tumors. Of course, this first and rather limited study of the antitumor activity of new polyfunctional compound T1084 leaves many open questions that require further detailed study. Firstly, its safety studies. It is known that DHA in some pathologies can cause peripheral neuropathy. In this regard, the toxicological characteristics of T1084 require detailed clarification in studies of cumulativeness, cytotoxicity, and tumor-specificity in vitro. No less significant is the further morphological, gistological, and immunohistochemical studies of toxic, antitumor, and antimetastatic effects in models close to common human tumors, including in combination with existing methods of radio- and chemotherapy. We have planned or have already begun studies in all these areas.

In conclusion, it should be noted that the approach used in this work potentially allows the implementation of other polyfunctional antitumor compounds with different molecular mechanisms of action. Thus, in particular, we managed to synthesize the salt of 1-isobutanoyl-2-isopropylisothiourea with 3-bromopyruvate [28], which also has synergistic antitumor properties [29]. However, it should be recognized that this compound is inferior to T1084 due to its rather high toxicity.

## 4. Materials and Methods

### 4.1. Animals

Male outbred ICR mice (CD-1; 3–4 months old, 25–28 g body weight), male and female F_1_(CBA × C57BL/6j) mice (3–4 months old, 22–25 g body weight), and male CBA mice (3–4 months old, 24–27 g body weight) were used in these studies. The animals were purchased from the Biomedical Technology Scientific Center of Federal Biomedical Agency of Russia (Moscow). The animals were housed in T-3 and T-4 cages under natural light conditions with forced ventilation 16 times/h, at a room temperature 18–20 °C and relative humidity 50–70%. The animals had free access to water and rodents PK-120-1 feed (Laboratorsnab Ltd., Moscow, Russia). Animal studies were approved by the A. Tsyb Medical Radiological Research Center (MRRC) Ethical Committee and were performed in accordance with generally accepted standards for the animal treatment based on standard operating procedures of the A. Tsyb MRRC in accordance with the rules and requirements of the European Convention ETS/STE No. 123 and international standard GLP (OECD Guide 1:1998).

### 4.2. T1084 and Other Compounds: Synthesis and Administration

The studied compound T1084—1-isobutanoyl-2-isopropylisothiourea dichloroacetate, and the substances used for a comparison—1-isobutanoyl-2-isopropylisothiourea hydrobromide (T1023) and sodium dichloroacetate (Na-DCA), were synthesized in the laboratory of radiation pharmacology A. Tsyb MRRC. T1023 was synthesized by the previously described method [30]. The synthesis of Na-DCA was carried out by the interaction of equimolar amounts of DCA and NaOH.

The method for T1084 preparing consisted in the isolation of the free base, 1-isobutanoyl-2-isopropylisothiourea, from the T1023 compound, which was then reacted with DCA [23]. The T1084 structure has been confirmed by NMR-spectrometry and elemental analysis. Spectra 1H NMR was obtained in DMSO-d6 on the spectrometer DRX-500 (Bruker, Germany) at a frequency of 500 MHz with tetramethylsilane as internal standard. Elemental analysis of C, H, and N was performed on an elemental analyzer EA 1108 (Carlo Erba Instruments, Italy). The control of T1084 purity was performed using thin layer chromatography and melting temperature measurements. Chromatography on Silufol UV-254 plates (Czech Republic) was performed in a benzene–ethanol–triethylamine (9:1:0.1) system. The melting point of T1084 was determined on an automatic heating unit PTP-M (LOIP Ltd., Russia). Synthesis example: 1.3 g of 1-isobutanoyl- 2-isopropylisothiourea hydrobromide was dissolved in 10 mL of water, a concentrated aqueous solution of ammonia was added dropwise to pH 8 and extracted twice with diethyl ether (2 × 10 mL). Dried with anhydrous Na_2_SO_4_ and then evaporated. Base yield 0.7 g. The resulting base was dissolved in 5 mL of diethyl ether, and a solution of 0.5 g of DCA in 3 mL of diethyl ether was added to it. Cooled, the precipitate was filtered off and recrystallized from hexane. Yield was 0.8 g of white crystalline T1084 (68%). Spectra 1H NMR (500 MHz; DMSO-d6, δ): 1.07 (d, 6H); 1.3 (d, 6H); 2.5 (m, 1H); 3.8 (b, 3H); 4.1 (m, 1H); 6.6 (s, 1H). Calculated (%): C 37.86; H 5.72; N 8.83. Found (%): C 37.91; H 5.69; N 8.87. The melting temperature is 67–69 °C. Rf value is 0.35. Molecular weight: 317.2 g/mol. Chemical formula: C_8_H_16_N_2_OS·C_2_H_2_Cl_2_O_2_.

In the study of toxic properties, NOS-inhibiting and antitumor activity, T1084, Na-DCA and T1023 were injected once, intraperitoneally (i.p.). In the study of PDK-inhibitory activity, Na-DCA and T1084 were administered 4 times intragastrically (i.g.). In all cases, all compounds were used in the form of aqueous solutions made *ex tempore* on the basis of water for injection (JSC Dalkhimpharm, Khabarovsk, Russia). All control animals were injected i.p. with an aseptic 0.9% sodium chloride solution (JSC Dalkhimpharm) in an equivalent volume according to similar schemes.

### 4.3. Toxicology

Acute toxicity studies were carried out using Litchfield-Wilcoxon method. Fifty white outbreed male mice were divided into 7 groups, 7–8 animals in each. T1084 was administered i.p. at doses of 150–600 mg/kg in 0.2 mL injection volume. Each animal was monitored individually for 15 days, with special attention given during the first 4 h.

### 4.4. NOS-Inhibitory Activity In Vivo

To compare the NOS inhibitory activity in vivo of T1023, Na-DCA and T1084, we studied the effect of these compounds at equimolar doses on the content of stable NO metabolites—nitrites and nitrates (NO_2_/NO_3_), in liver tissues of male mice F_1_(CBA × C57BL6j) induced by lipopolysaccharide (LPS) *E. coli* (O111: B4; Sigma-Aldrich, St. Louis, MO, USA). 

Then, 60 mice were divided into 7 groups—control and 6 experimental groups, 8–9 animals in each. At the beginning of the experiment, the animals of all experimental groups were injected once i.p. with LPS at a dose of 4 mg/kg in 0.2 mL of solution, and control animals with 0.2 mL of 0.9% sodium chloride solution. After 4 h, the animals of the second and third experimental groups were injected once with T1023 or T1084 i.p. in equimolar doses of 37 μM/kg (10.0 and 11.8 mg/kg, respectively), and the animals of the fourth, fifth, and sixth groups were injected once with T1023, Na-DCA, or T1084, i.p. in equimolar doses 148 µM/kg (40.0, 22.2 and 47.1 mg/kg, respectively). According to available data [30], T1023 at such doses exhibits a long-term (3–6 h) NOS inhibitory effect in vivo, which should affect the content of NO_2_/NO_3_ in tissues. After 24 h from the beginning of the manipulations, all animals were euthanized. The liver was removed, and homogenates were prepared in PBS on ice in a short time. From the homogenate of each organ, 3 samples were prepared for measurements. Samples were deproteinized with zinc sulfate, reduction in nitrates to nitrites was carried out with metallic zinc. The nitrite content in the samples was measured by the amperometric method in a reducing solution of H_2_SO_4_ using an ISO-NOP 2mm NO-selective electrode (WPI, Sarasota, FL, USA).

### 4.5. PDK- Inhibitory Activity In Vivo

A comparative study of the effect of Na-DCA, T1084, and T1023 at equimolar doses on PDK activity was carried out by measuring the rate of pyruvate oxidation in male CBA mouse liver tissues. 

70 mice were divided into 6 groups—control and 5 experimental groups, 11–13 animals in each. At the beginning of the experiment, the animals of 1–4 experimental groups were administered 4 times, with an interval of 6 h, Na-DCA or T1084 i.g. in equimolar doses of 200 μM/kg (30 and 63 mg/kg, respectively) and 330 μM/kg (50 and 105 mg/kg, respectively) in 0.2 mL of solution. The animals of the 5th group received T1023 i.g., 330 μM/kg (89 mg/kg). Control mice during these periods received i.g. 0.2 mL of 0.9% sodium chloride solution. Then, 3 h after the last injection, the animals were euthanized, and the liver was removed. A homogenate was prepared from 1 g of liver tissue in a short time on ice, from which 4 samples were prepared for measurements.

The oxidation rate of pyruvate in prepared samples was measured by a ferricyanide spectrophotometric assay for measuring pyruvate dehydrogenase complex activity [31]. The reaction was initiated by adding 0.1 mL of 0.4 M sodium pyruvate (PanEco, Moscow, Russia) and 0.4 mL of liver tissue homogenate to the reaction mixture. After 40 min of incubation at 37 °C, the reaction was stopped, the mixture was centrifuged, and the extinction at 417 nm in the supernatant was measured using a Unico-2100 spectrophotometer (United Products & Instruments, Dayton, NJ, USA).

### 4.6. Anti-Tumor Activity In Vivo

T1084 antitumor activity was studied using SEC model in mice. The SEC strain was obtained from the tumor bank N.N. Blokhin National Medical Research Center of Oncology of the Ministry of Health of Russian Federation. Then, 2.5 × 10^6^ tumor cells in 0.3 mL of medium 199 (Pan-Eco, Moscow, Russia) were transplanted s/c into the area of the lateral surface of the right thigh in F_1_(CBA × C57BL/6j) female mice.

Two independent experiments were carried out according to the same scheme. After neoplasia inoculation, the animals were divided into 4 groups—control and three experimental groups, 20–21 mice in each. Experimental treatment began on the 6th–8th day after inoculation, when the SEC tumor nodes in all individuals reached a reliably measurable size—60–80 mm^3^. From this day until the 20th day of tumor growth, mice of the experimental groups were injected daily with T1023, Na-DCA, or T1084 at equimolar doses of 220 µM/kg (60.0, 33.6 and 70.7 mg/kg, respectively) in 0.2 mL of solution. Animals of the control group were injected daily with 0.2 mL of 0.9% sodium chloride solution. Duration of experiments was 21–25 days from the tumor transplantation.

The development of neoplasia in the experiments was assessed morphometrically. The sizes of tumor nodes in all mice were measured every 2–3 days with a caliper, and their volumes were estimated in approximation V=a·b·c·(π/6), where a, b, and c are orthogonal diameters. Then, the relative volumes of neoplasias were calculated by normalizing the tumor volume in each animal on the day of the beginning of the exposure, as well as the indices of inhibition of tumor growth in the treated animals: $TI_{i,t}=\left({\bar{V}}_{C,t}-V_{i,t}\right)/{\bar{V}}_{C,t}\times 100\%$, where $\;TI_{i,t}$—inhibition index in the *i*th animal at the time of observation *t*; ${\bar{V}}_{C,t}$—the average relative tumor volume in the control at the time of observation *t*; and $V_{i,t}$—relative tumor volume of the *i*th animal at the time of observation *t*.

The severity and dynamics of T1023, Na-DCA, and T1084 antitumor effect were assessed and compared by means of multiple intergroup statistical comparison of neoplasia relative volumes and indicators of tumor growth inhibition at different periods of observation. The integral antitumor effect was assessed by the duration of the SEC growth inhibition. The time of a 10-fold increase in volume on the curves of tumor growth was estimated, as well as the difference in the mass of tumor nodes isolated at the end of the observation.

### 4.7. Statistical Analysis

The standard parameters of the variation statistics were calculated for all experimental data. Their values are given, including graphically, in the form M ± SD. For paired comparisons, the level of significance of intergroup differences in indicators was assessed using the Mann–Whitney U test for multiple comparisons using the Kruskal–Wallis ANOVA of ranks with the post hoc Mann–Whitney U test by Bonferroni–Holm multiple test procedure. In all cases, the effects and differences were considered statistically significant at the 5% level. Statistical calculations were performed using the software package Statistica 10.0 (StatSoft Inc., Tulsa, OK, USA).

## 5. Conclusions

The proposed chemical approach made it possible to synthesize a bifunctional compound able to simultaneously exhibit NOS-inhibiting and PDK-inhibiting activities. Studies on the SEC model in mice indicate that it is capable of not only exerting a synergistic antitumor effect, but also suppressing the development of neoplasia resistance.

The totality of the data obtained indicates that the combination of antiangiogenic and hypoxia-oriented toxic effects (in this case, within the molecular structure of the active substance) can be a promising direction for improving malignant neoplasm chemotherapy.

## Figures and Tables

**Table 1:** T1084 and T1023 acute toxicity for male outbred mice, single i.p. administration.

Compound	LD_16_		LD_50_		LD_84_	
	mg/kg	mM/kg	mg/kg	mM/kg	mg/kg	mM/kg
T1084	330	1.04	447 ± 13	1.41	613	1.93
T1023 *	272	1.01	410 ± 17	1.52	552	2.04

Note. *—indicators of acute toxicity of compound T1023 obtained earlier [11,12].

## Abbreviations

NO	endogenous nitric oxide
NOS	nitric oxide synthase
PDH	pyruvate dehydrogenase
PDK	pyruvate dehydrogenase kinase
DCA	dichloroacetates
SEC	solid Ehrlich carcinoma

## References

[1] Miller T.W., Isenberg J.S., Roberts D.D. (2009). Molecular regulation of tumor angiogenesis and perfusion via redox signaling. Chem. Rev..

[2] Ziche M., Morbidelli L. (2009). Molecular regulation of tumor angiogenesis by nitric oxide. Cytokine Netw..

[3] Rabender C.S., Alam A., Sundaresan G., Cardnell R.J., Yakovlev V.A., Mukhopadhyay N.D., Graves P., Zweit J., Mikkelsen R.B. (2015). The role of nitric oxide synthase uncoupling in tumor progression. Mol. Cancer Res..

[4] Marech I., Leporini C., Ammendola M., Porcelli M., Gadaleta C.D., Russo E., De Sarro G., Ranieri G. (2016). Classical and non-classical proangiogenic factors as a target of antiangiogenic therapy in tumor microenvironment. Cancer Lett..

[5] Vahora H., Khan M.A., Alalami U., Hussain A. (2016). The potential role of nitric oxide in halting cancer progression through chemoprevention. J. Cancer Prev..

[6] Kashiwagi S., Izumi Y., Cohongi T., Demou Z.N., Xu L., Huang P.L., Buerk D.G., Munn L.L., Jain R.K., Fukumura D. (2005). NO mediates mural cell recruitment and vessel morphogenesis in murine melanomas and tissue-engineering blood vessels. J. Clin. Investig..

[7] Mohamad N.A., Cricco G.P., Sambuco L.A., Croci M., Medina V.A., Gutierrez A.S., Bergoc R.M., Rivera E.S., Martin G.A. (2009). Aminoguanidine impedes human pancreatic tumor growth and metastasis development in nude mice. World J. Gastroenterol..

[8] Lampson B.L., Kendall S.D., Ancrile B.B., Morrison M.M., Shealy M.J., Barrientos K.S., Crowe M.S., Kashatus D.F., White R.R., Gurley S.B. (2012). Targeting eNOS in pancreatic cancer. Cancer Res..

[9] Janakiram N.B., Rao C.V. (2012). iNOS-selective inhibitors for cancer prevention: Promise and progress. Future Med. Chem..

[10] Gao Y., Zhou S., Xu Y., Sheng S., Qian S.Y., Huo X. (2019). Nitric oxide synthase inhibitors 1400W and L-NIO inhibit angiogenesis pathway of colorectal cancer. Nitric Oxide.

[11] Filimonova M.V., Shevchenko L.I., Makarchuk V.M., Shevchuk A.S., Juzhakov V.V., Tsyb A.F. (2014). Medical Radiological Research Center of RAMS. Anticancer Agent.

[12] Filimonova M.V., Yuzhakov V.V., Shevchenko L.I., Bandurko L.N., Sevankaeva L.E., Makarchuk V.M., Chesnakova E.A., Shevchuk A.S., Tsyganova M.G., Fomina N.K. (2015). Experimental study of antitumor activity of new nitric oxide synthase inhibitor T1023. Mol. Med..

[13] Filimonova M.V., Yuzhakov V.V., Filimonov A.S., Makarchuk V.M., Bandurko L.N., Korneeva T.S., Samsonova A.S., Tsyganova M.G., Shevchenko L.I., Sevankaeva L.E. (2019). Comparative study of the effects of NOS inhibitor T1023 and bevacizumabum on growth and morphology of Lewis lung carcinoma. Phatol. Physiol. Exp. Ther..

[14] Filimonova M.V., Makarchuk V.M., Shevchenko L.I., Filimonov A.S. (2019). Effect of a NOS inhibitor T1023 in combination with γ-irradiation and cyclophosphamide on growth and metastasis of Lewis lung carcinoma. Phatol. Physiol. Exp. Ther..

[15] Frandsen S., Kopp S., Wehland M., Pietsch J., Infanger M., Grimm D. (2016). Latest results of anti-angiogenic drugs in cancer treatment. Curr. Pharm. Des..

[16] Zirlik K., Duyster J. (2018). Anti-angiogenics: Current situation and future perspectives. Oncol. Res. Treat..

[17] Koblyakov V.A. (2014). Hypoxic state and glycolysis as a possible anticancer therapeutic target. Adv. Mol. Oncol..

[18] Zhao Y., Liu H., Liu Z., Ding Y., Ledoux S.P., Wilson G.L., Voellmy R., Lin Y., Lin W., Nahta R. (2011). Overcoming transtuzumab resistance in breast cancer by targeting dysregulated glucose metabolism. Cancer Res..

[19] Shen Y.C., Ou D.L., Hsu C., Lin K.L., Chang C.Y., Lin C.Y., Lin S.H., Cheng A.L. (2013). Activating oxidative phosphorylation by pyruvate dehydrogenase kinase inhibitor overcomes sorafenib resistance of hepatocellular carcinoma. Br. J. Cancer.

[20] Kumar K., Wigfield S., Gee H.E. (2013). Dichloroacetate reverses the hypoxic adaptation to bevacizumab and enhances its antitumor effects in mouse xenografts. J. Mol. Med..

[21] Filimonova M.V., Podosinnikova T.S., Samsonova A.S., Makarchuk V.M., Shevchenko L.I., Filimonov A.S. (2019). Comparison of antitumor effects of combined and separate treatment with NO synthase inhibitor T1023 and PDK1 inhibitor dichloroacetate. Bull. Exp. Biol. Med..

[22] Filimonova M.V., Korneeva T.C., Shevchenko L.I., Samsonova A.S., Filimonov A.S. (2019). PDK suppression due to chronic influence of NOS inhibitor blocks the development of hypoxic resistance of experimental neoplasions. Cell Death Discov..

[23] Filimonova M.V., Shevchenko L.I., Filimonov A.S., Korneeva T.S., Samsonova A.S. (2019). National Medical Research Center of Radiology of Ministry of Health of the Russian Federation. Agent for Targeted Therapy of Malignant Growths.

[24] Bellou S., Pentheroudakis G., Murphy C., Fotsis T. (2013). Anti-angiogenesis in cancer therapy: Hercules and hydra. Cancer Lett..

[25] Mahase S., Rattenni R.N., Wesseling P., Leenders W., Baldotto C., Jain R., Zagzag D. (2017). Hypoxia-mediated mechanisms associated with antiangiogenic treatment resistance in glioblastomas. Am. J. Pathol..

[26] Itatani Y., Kawada K., Yamamoto T., Sakai Y. (2018). Resistance to anti-angiogenic therapy in cancer—alterations to anti-VEGF pathway. Int. J. Mol. Sci..

[27] Terry S., Zaarour F.R., Venkatesh H.G., Francis A., El-Sayed W., Buart S., Bravo P., Thiery J., Chouaib S. (2018). Role of hypoxic stress in regulating tumor immunogenicity, resistance and plasticity. Int. J. Mol. Sci..

[28] Filimonova M.V., Shitova A.A., Soldatova O.V., Shevchenko L.I., Filimonov A.S., Podosinnikova T.S., Saburova A.S. (2021). National Medical Research Radiological Center of the Ministry of Health of the Russian Federation. Complex Anti-Tumoral Product.

[29] Shitova A.A., Soldatova O.V., Filimonova M.V., Shevchenko L.I., Saburova A.S., Podosinnikova T.S., Filimonov A.S. (2020). Estimation of antitumor activity of compound T1097—NOS inhibitor and glycolysis inhibitor—On experimental Erlich carcinoma in vivo. J. Phys. Conf. Ser..

[30] Filimonova M.V., Makarchuk V.M., Shevchenko L.I., Saburova A.S., Surinova V.I., Izmestieva O.S., Lychagin A.A., Saburov V.O., Shegay P.V., Kaprin A.D. (2020). Radioprotective activity of the nitric oxide synthase inhibitor T1023. toxicological and biochemical properties, cardiovascular and radioprotective effects. Radiat. Res..

[31] Bisswanger H. (2013). Practical Enzymology.

